# Rice husk-SiO_2_ supported bimetallic Fe–Ni nanoparticles: as a new, powerful magnetic nanocomposite for the aqueous reduction of nitro compounds to amines

**DOI:** 10.1039/d0ra05381c

**Published:** 2020-09-10

**Authors:** Mohammad Ghadermazi, Setareh Moradi, Roya Mozafari

**Affiliations:** Department of Chemistry, University of Kurdistan P.O. Box 66135-416 Sanandaj Iran mghadermazi@yahoo.com +98 87 3324133 +98 87 33624133

## Abstract

This paper reports a novel green procedure for immobilization of bimetallic Fe/Ni on amorphous silica nanoparticles extracted from rice husk (RH-SiO_2_). The heterogeneous nanocomposite (Fe/Ni@RH-SiO_2_) was identified using SEM, EDX, TEM, BET, H_2_-TPR, TGA, XRD, VSM, ICP-OES, and FT-IR analyses. The Fe/Ni@RH-SiO_2_ nanocomposite was applied as a powerful catalyst for the reduction of structurally diverse nitro compounds with sodium borohydride (NaBH_4_) in green conditions. This procedure suggests some benefits such as green chemistry-based properties, short reaction times, non-explosive materials, easy to handle, fast separation and simple work-up method. The catalyst was separated by an external magnet from the reaction mixture and was reused for 9 successive cycles with no detectable changes of its catalytic efficiency.

## Abstract 2

This paper reports a novel green procedure for immobilization of bimetallic Fe/Ni on amorphous silica nanoparticles extracted from rice husk (RH-SiO_2_).

## Graphical abstract

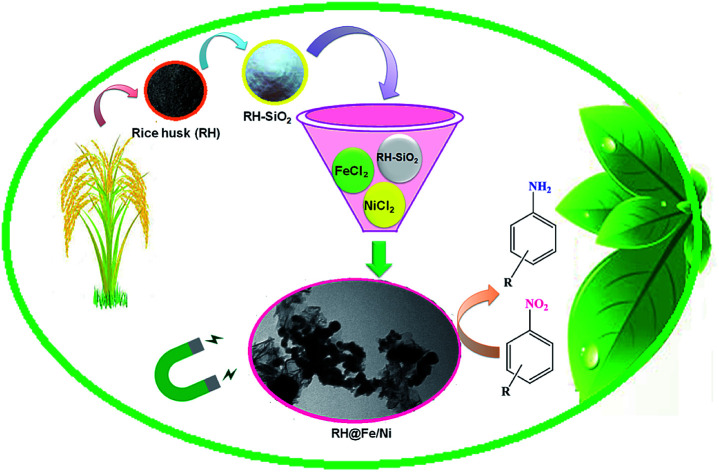

## 1. Introduction

Agricultural wastes are defined as the residues from the growing and processing of raw agricultural products such as fruits, vegetables, and crops. Utilization of agricultural processing wastes in the production of high value-added products has increased the profitability of the food processing industry by reducing the cost of disposal of these wastes.^1–3^ Rice husk is one of the most valuable agricultural wastes in the world.^4^ As it contains over 70% silica and can be an economically viable raw material for the preparation of several silicon compounds, including sodium silicate,^5^ silicon nitride,^6^ silicon carbide,^7^ pure silicon and zeolite.^8^ The inorganic silica possesses considerable advantages such as abundant availability, adequate surface functionalization, hydrophilic nature, sustainable and almost free of cost, unique biocompatibility of silanol (Si–OH) and silicate skeletons (Si–O–Si).^9^ The amorphous silica with high surface area can be extracted from the RH by chemical treatment with acid followed by process of burning at 700 °C.^10^

The rice husk can be modified by changing of the grafted species. Transition metals incorporated onto the surface of RH-SiO_2_ act as an efficient catalyst in several reactions, such as oxidation,^11^ acylation,^12^ benzylation,^13^*etc.* Therefore, several supported metal catalysts have been recently prepared and their applications have been developed in the organic reactions. Especially, metal-containing organosilica nanocomposite is more interested due to its remarkable capacity in catalytic processes.^14,15^

Besides, nickel catalysis has made tremendous advances in organic synthesis, especially the synthesis of heterocycle compounds, which have been widely used among a variety of pharmaceutical compounds due to their relative insensitivity to air, low toxicity and price.^16–20^

The design and preparation of green catalysts have attracted much attention in both academic area and pharmaceutical industry.^21^ For this mean, heterogenization of the catalysts to solid supports is one of the interesting choices to prevent wasting the catalyst. Besides, the supports usually have a great effect on the activity of the catalyst.^22,23^ The heterogeneity of catalysts to solid bases can provide opportunities for the easy separation of the catalysts from reaction mixtures.^24–26^ Also, current literature reveals that the catalytic activity of Ni nanoparticles is high but their small nano-sized particles cannot be separated by conventional methods such as filtration.^27–29^ Therefore, nickel is loaded on the surface or inside the pores of solid supports such as zeolite, cellulose, silica and polymer, which could be useful way to fix the mentioned shortcomings.^30,31^ A great deal of attention has been recently devoted to metal catalysts such as CoFe_2_O_4_@GO–K 22·Ni,^32^ Ni/Mg@MCM-41,^33^ Cu–Ni/Ti-MCM-41,^34^ Fe_3_O_4_@PMDA/Pd,^35^ NiCu/SBA15 ^36^ and SBA-15@ABA-M (M = Cu, Ni and Pd).^37^

Aromatic nitro compounds are highly toxic and are introduced to the environment through the wastewaters of dyes, agrochemicals, synthetic resins and plastics *etc.*^38–43^ On the other hand, the nitroarenes reduced to arylamines are considered as important starting materials and intermediates in pharmaceutical, antioxidants and agricultural industries.^44–47^ Therefore, the catalytic reduction of nitro compounds to amines in sustainable method with green solvent has attracted a great deal of attention. Some catalytic systems have been reported such as γ-Fe_2_O_3_@HAp-CD·Ag,^48^ Fe_3_O_4_@TA/Ag,^49^ NiFe_2_O_4_@Cu,^50^ Co_3_O_4_-NGr/C,^51^ Fe_3_O_4_@SiO_4_,^52^ Pd-NPs@G.Tea,^53^ Fe_3_O_4_@Cu(OH)_*x*_/NaBH_4_,^54^ RGO@Pt/H_2_,^55^ CuFe_2_O_4_/NaBH_4_.^56^ For further reviews on heterogeneous catalytic reduction, see ref. 57–62.

In spite of their merits, the most commonly reported methods suffer from the use of hazardous reagents, unsatisfactory yields, environmental pollution, large amounts, poor recovery of expensive catalysts and long reaction times, which ultimately lead to the production of large amount of toxic waste. With the purpose of developing more convenient or selective catalysts or reagents, reducing the number of separate reaction steps, higher efficiency and minimal by-products, herein, for the first time a novel magnetically retrievable nanocomposites of RH-SiO_2_ and Fe/Ni is prepared, characterized and its catalytic system application for reduction of various nitro groups to the corresponding amines with NaBH_4_ as reducing agent in H_2_O is investigated. The synthesized nanocatalyst with a combination of the catalytic activity of Ni nanoparticles and the magnetic properties of Fe is shown to have an excellent catalytic activity, as well as the recoverability and reusability in the synthesis of amines.

## 2. Experimental

### 2.1 Characterization methods

The instruments used for the characterization of Fe/Ni@RH-SiO_2_ include FTIR, TGA, FESEM, EDX, TEM, XRD, V SM, ICP-OES, BET, H_2_-TPR. The models of the apparatus are as following: the particle morphology and size were examined by measuring scanning electron microscopy (SEM) using SEM-TESCAN MIRA3. Energy-dispersive X-ray spectroscopy (EDX) analysis was obtained by MIRA3TESCANXMU instrument. Thermogravimetric analysis (TGA) was determined by Shimadzu DTG-60 instrument. The X-ray powder diffraction (XRD) data were collected by Co Kα radiation (*λ* = *n*1.78897 Å) operating at *n*40 keV. Vibrating sample magnetometer (VSM) measurement was recorded by a Vibrating Sample Magnetometer MDKFD. The content of cobalt and vanadium in the nanocomposite were measured by inductively coupled plasma-optical emission spectrometry (ICP-OES). H_2_-Temperature Programmed Reduction (H_2_-TPR) experiment were carried out using a NanoSORD NS91 (made by Sensiran Co., Iran) apparatus. Fourier transforms infrared (FT-IR) spectra were investigated by KBr disc using a VRTEX 70 model BRUKER FT-IR spectrophotometer. The size of the as-synthesized nanoparticle was determined by transmission electron microscopy (TEM) techniques using Zeiss-EM10C Transmission electron. The specific surface area and the pore size distribution of nitrogen uptake pores at 77.4 K on a Belsorp18, were calculated using the Brunauer–Emmett–Teller method and the Barrett–Joyner–Halenda model, respectively. NMR spectra were taken using a BRUKER Advance 400 MHz NMR spectrometer and deuterated DMSO.

### 2.2 Production of silica from residual rice husk

First, 30 g of residual rice husk with 1.0 M nitric acid was stirred in a 250 mL flask at room temperature for approximately 24 h. It was rinsed thoroughly with distilled water until the pH of the solution is fixed, then it was dried at 80 °C in the oven, and was incinerated in the muffle oven for 6 h at 600–800 °C to turn white. To extract silica from rice husk, 5 g of ash was stirred in 250 mL of 1.0 M sodium hydroxide solution at room temperature for 24 h. Then the combination was filtered and titrated with 3.0 M nitric acid. Titration (the drop in HNO_3_ was controlled by a dropper with a drop of 1 mL min^−1^) and continued until the pH of the solution reached 5.0. Next, smooth silica gel was washed with distilled water, and dried at 100 °C for 24 hours in the oven.

### 2.3 Preparation of Fe@RH-SiO_2_ nanocomposite

In a 250 mL 3-necked, round-bottomed flask, 1 g of nanosilica (extracted from rice husk) was ultrasonicated in 50 mL of distilled water for 30 minutes. A solution of iron(ii) chloride (1.78 g in distilled water 25 mL) was added to the suspension of nanosilica and was ultrasonicated for 30 minutes and the mixture was stirred for 30 minutes in the N_2_ atmosphere. Afterwards, a solution of sodium borohydride (1.35 g in distilled water 50 mL) was added dropwise with constant stirring under inert atmosphere, the black Fe nanoparticles were immobilized on RH-SiO_2_ nanospheres. Ultimately, the final product was isolated by an external magnet and washed twice with distilled water/ethanol and dried in oven at 70 °C for 12 h.

### 2.4 Preparation of Fe/Ni@RH-SiO_2_ nanocomposite

Briefly, in a 250 mL 3-necked, round-bottomed flask, Fe@RH-SiO_2_ (1 g), was sonicated in 30 mL of deionized water for 20 min. Then a solution of NiCl_2_·2H_2_O (0.8 g, 6 mmol) in deionized water (30 mL) was added to the suspension of Fe@RH-SiO_2_ and the mixture was again sonicated for 20 min. Subsequently, 0.27 g of sodium borohydride was dissolved in 30 mL of H_2_O and then was added dropwise into the above reactant mixtures and was stirred for 1 h at room temperature. In the next step, the composite was separated by an external magnet and washed with ethanol/deionized water several times. Black solid of Fe/Ni@RH-SiO_2_ nanocomposite was dried overnight at room temperature before use.

### 2.5 A general procedure for reduction of nitro compounds with NaBH_4_ in the presence of Fe/Ni@RH-SiO_2_ nanocomposite

In a 50 mL round bottom flask equipped with a magnetic stirrer, nitro compound (1 mmol), H_2_O (3 mL) and Fe/Ni@RH-SiO_2_ (0.03 g) were added and the mixture was stirred for 1 min at room temperature. Then, NaBH_4_ (4 mmol, 0.151 g) was added to the flask and the mixture was stirred vigorously at 50 °C. After completion of the reaction as indicated by TLC [using ethyl acetate/*n*-hexane as eluent: 1/5], the insoluble supported nanocatalyst was isolated with the aid of an external magnetic and the product was extracted from liquid with diethyl ether. The products were purified by column chromatography.

Products 1–11 are known compounds, and their NMR spectra are in accordance with those reported in the literature.^63–68^

#### Aniline (1)

Yellow oil. Evaporation of the solvent under reduced pressure affords a crude product that is purified by column chromatography. ^1^H NMR (400 MHz, CDCl_3_) *δ* (ppm) = 7.16 (m, 2H, ArH), 6.77 (m, 1H, ArH), 6.69 (m, 2H, ArH), 3.35 (br. s, 2H, NH_2_).

#### Benzene-1,4-diamine (2)

Light brown solid, mp = 141–143 °C. Evaporation of the solvent under reduced pressure affords a crude product that is purified by recystallization from EtOH to yield 2. ^1^H-NMR (400 MHz, CDCl_3_) *δ* (ppm) = 4.18 (s, 4H), 6.36 (s, 4H).

#### 4-Aminophenol (3)

Brown solid, mp = 188–190 °C. Evaporation of the solvent under reduced pressure affords a crude product that is purified by recystallization from EtOH to yield 3. ^1^H NMR (400 MHz, DMSO) *δ* (ppm) = 6.45 (d, *J* = 8.56 Hz, 2H, ArH), 6.38 (d, *J* = 8.56 Hz, 2H, ArH), 4.35 (br. s, 2H, NH_2_).

#### Benzen-1,2-diamine (4)

Light brown solid, mp = 105–108 °C. The product was purified by column chromatography. ^1^H NMR (400 MHz, CDCl_3_) *δ* (ppm) = 6.73–6.69 (m, 4H), 3.29 (s, 4H).

#### 4-Chloroaniline (5)

Pale yellow oil. The product was purified by column chromatography. ^1^H NMR (400 MHz, CDCl_3_) *δ* (ppm) = 7.13–7.06 (m, 1H), 6.64–6.56 (m, 1H), 3.65 (s, 1H).

#### 4-(Aminophenyl)methanol (6)

Pale yellow solid, mp = 82 °C. The product was purified by column chromatography. ^1^H NMR (400 MHz, CDCl_3_) *δ* (ppm) = 7.03 (d, *J* = 8.1 Hz, 2H, Ar), 6.61 (d, *J* = 8.4 Hz, 2H, Ar), 4.42 (s, 2H, CH_2_), 2.94 (bs, 3H, NH_2_ and OH).

#### p-Toluidine (7)

Yellow oil. The product was purified by column chromatography. ^1^H NMR (400 MHz, CDCl_3_) *δ* (ppm) = 6.97 (d, *J* = 7.0 Hz, 2H), 6.61 (d, *J* = 3.2 Hz, 1H), 6.59 (s, 1H), 3.51 (s, 2H), 2.24 (d, *J* = 6.2 Hz, 3H).

#### 4-(1-Hydroxyethyl)aniline (8)

Yellow solid, mp = 93–95 °C. The product was purified by column chromatography. ^1^H NMR (400 MHz, CDCl_3_) *δ* (ppm) = 7.89–7.76 (m, 2H), 6.64 (d, *J* = 8.5 Hz, 2H), 4.16 (s, 2H), 2.77 (bs, 1H) 1.51 (s, 3H).

#### 4-Aminobenzoicacid (9)

Light pink solid, mp = 181 °C. Evaporation of the solvent under reduced pressure affords a crude product that is purified by recystallization from EtOH to yield 9. ^1^H NMR (400 MHz, CDCl_3_) *δ* (ppm) = 7.92 (t, *J* = 2.2 Hz), 7.9–7.89 (m), 6.68–6.63 (m), 4.12 (s).

#### Butan-1-amine (10)

Yellow oil. The product was purified by column chromatography. ^1^H NMR (400 MHz, CDCl_3_) *δ* (ppm) = 2.56 (t, *J* = 6.9 Hz, 2H), 1.75 (s, 2H), 1.37–1.15 (m, 4H), 0.79 (t, *J* = 7.1 Hz, 3H).

#### Propan-1-amine (11)

Pale yellow oil. The product was purified by column chromatography. ^1^H NMR (400 MHz, CDCl_3_) *δ* (ppm) = 2.60 (t, *J* = 6.2 Hz, 2H), 1.50 (m, 2H), 1.25 (s, 2H), 0.95 (t, *J* = 7.0 Hz, 3H).

### 2.6 A general procedure for the recovery of the Fe/Ni@RH-SiO_2_ nanocomposite

The reusability of the catalyst was evaluated by the reaction of nitrobenzene, H_2_O and NaBH_4_ using catalyst Fe/Ni@RH-SiO_2_ under optimized conditions (Table 2, entry 6). The catalytic activity of the recovered catalyst in the same reaction under optimized conditions shows that the magnetically separated nanocatalyst is reusable for nine consecutive runs without any significant loss in catalytic activity (Fig. 8).

## 3. Results and discussions

**Scheme 1:**
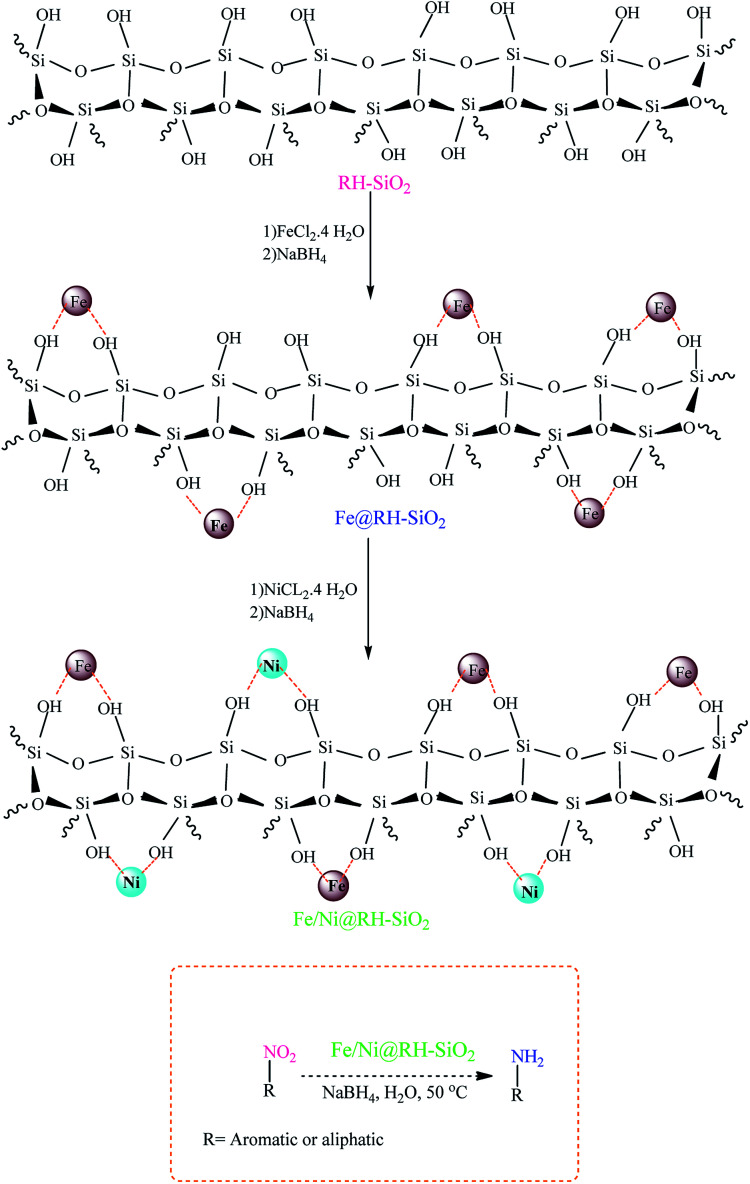
The schematic pathway for preparing Fe/Ni@RH-SiO_2_ nanocomposite and its application in the reduction of nitro compounds to amines.

The organic-inorganic Fe/Ni@RH-SiO_2_ nanocomposite was prepared with a three-step procedure (Scheme 1). For this goal, nano-silica was easily extracted from rice husk and the reaction was carried out by chemical reduction of Fe^2+^ and Ni^2+^ ions by NaBH_4_ to bimetallic Fe/Ni nanoparticles on the surface or inside the pores of RH-SiO_2_. For this propose, the supported Fe(0) on nano-silica (Fe@RH-SiO_2_) was primarily synthesized by the anchoring Fe nanoparticles on RH-SiO_2_. At the third step, deposition of Ni nanoparticles on Fe@RH-SiO_2_ afforded the nanocomposite of Fe/Ni@RH-SiO_2_. After that, the performance of the synthesized nanocomposite was assessed by applying it in the reduction of nitro compounds to corresponding amines in the presence of NaBH_4_. All reduction reactions were carried in water as green solvent at 50 °C (Scheme 1).

Characteristics of the nanocomposite, and the immobilization of bimetallic Fe/Ni on the pore surface of amorphous nanosilica was comfirmed using TEM and SEM images, XRD, TGA, ICP-OES, EDX, BET, FT-IR, VSM, and then reaction conditions were optimized.

### 3.1 Catalyst characterization

**Fig. 1:**
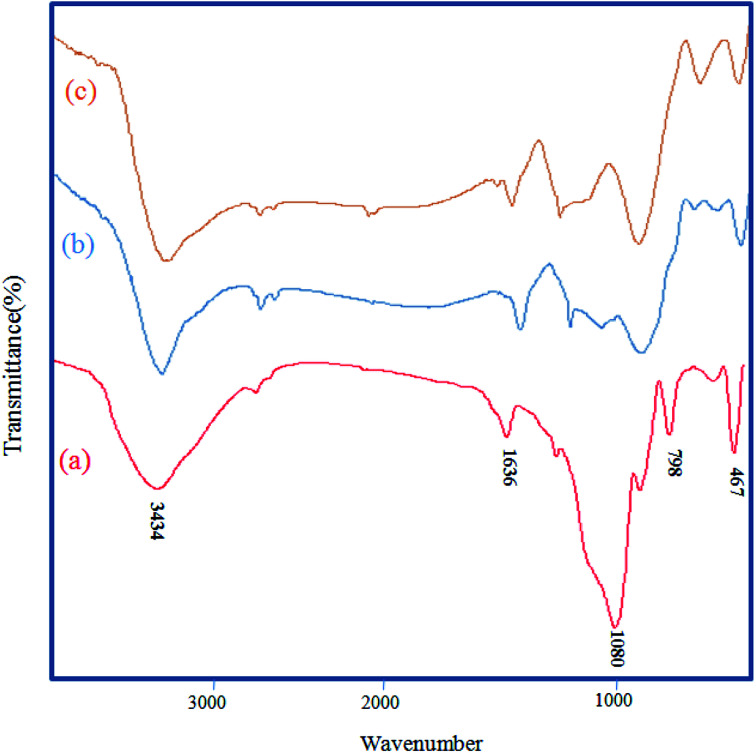
FT-IR spectra of RH-SiO_2_ (a), Fe@RH-SiO_2_ (b), and Fe/Ni@RH-SiO_2_ (c).

The FT-IR techniques are able to characterize and confirm the preparation of the catalyst. Fig. 1 demonstrates the FT-IR spectra of RH-SiO_2_ (a), Fe@RH-SiO_2_ (b), and Fe/Ni@RH-SiO_2_ (c). Spectrum of the RH-SiO_2_ (Fig. 1a) clearly exhibits absorption band at around 3434 cm^−1^ which is characteristic of the presence of stretching band of Si–OH bond and hydroxyl functional group (O–H) of water molecules adsorbed on the silicon oxide surface. The adsorption band at 1636 cm^−1^ can be attributed to the stretching of water molecules bound to the silica matrix. The peak at 1080 cm^−1^ is due to asymmetric stretching vibration of the structural Si–O–Si bond.^52^ The bands at 798 cm^−1^ and 467 cm^−1^ can be attributed to the deformation of Si–O.

The stretching of the Fe–O and Ni–O bands were observed at around 400–800 cm^−1^ which overlap with Si–O bonds. Additionally, the spectral analysis shows that a, b and c are the same, however, immobilization of bimetallic Fe/Ni on nanosilica (Fig. 1b and c) makes the peaks overlap and reduce their intensity.

**Fig. 2:**
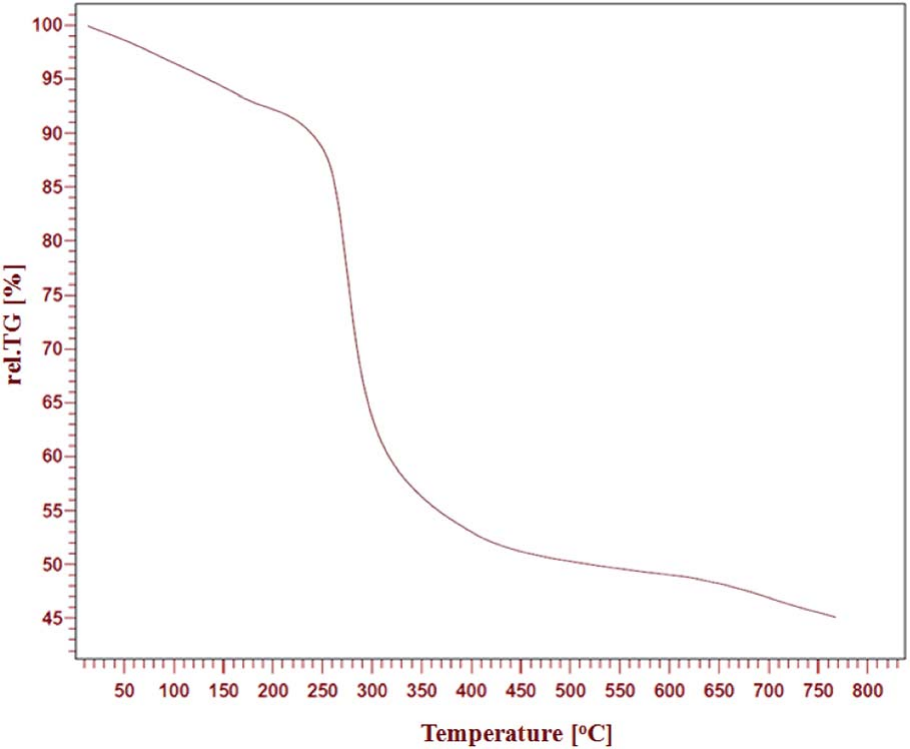
TGA curve of Fe/Ni@RH-SiO_2_ nanocomposite.

The thermal stability of Fe/Ni@RH-SiO_2_ was investigated by thermo-gravimetric analysis (TGA) in the temperature range of 25–800 °C (Fig. 2). One step of weight loss in the combined TGA curve shows that the magnetic nanocomposite has a reasonable stability up to 330 °C. Above this temperature, weight loss is serious but it could also indicate the parsing of RH-SiO_2_ structure.

These analyses entirely displayed that the heterogeneous system could be utilized successfully around mentioned temperature.

**Fig. 3:**
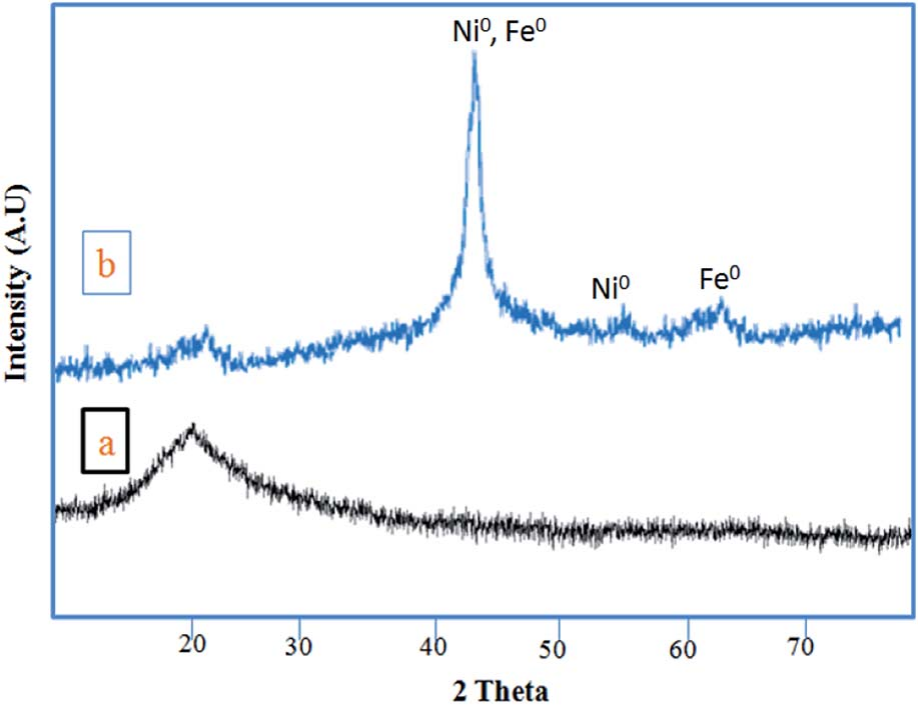
XRD patterns of RH-SiO_2_ (a) and Fe/Ni@RH-SiO_2_ (b).

Fig. 3 shows the XRD patterns of RH-SiO_2_ (a) and Fe/Ni@RH-SiO_2_ (b). In XRD pattern of the amorphous silica only one peak appeared at 2*θ* = 21.7 (Fig. 3a). The two reflections peaks at 2*θ* = 44.4° and 53.7°, which are related to (111) and (200) planes of metallic nickel with a face centered cubic structure. In addition, two peaks appeared at 2*θ* = 45.03° and 65.37° are assigned to Fe(0) (JCPDS 851410) (Fig. 3b). The absence of peaks at 35.6° and 38.5°, corresponding to Fe_2_O_3_, and Fe_3_O_4_, suggests that iron oxides were not formed.^69^

In XRD pattern of the amorphous silica only one peak appeared at 2*θ* = 21.7 (Fig. 3a). Retaining the characteristic peaks of RH-SiO_2_ after the anchoring of Fe/Ni reveals that the well-ordered structure of RH-SiO_2_ has not been destroyed, but two peaks appeared at 2*θ* = 45.03° and 65.37° are assigned to Fe(0) (JCPDS 851410) (Fig. 3b).

**Fig. 4:**
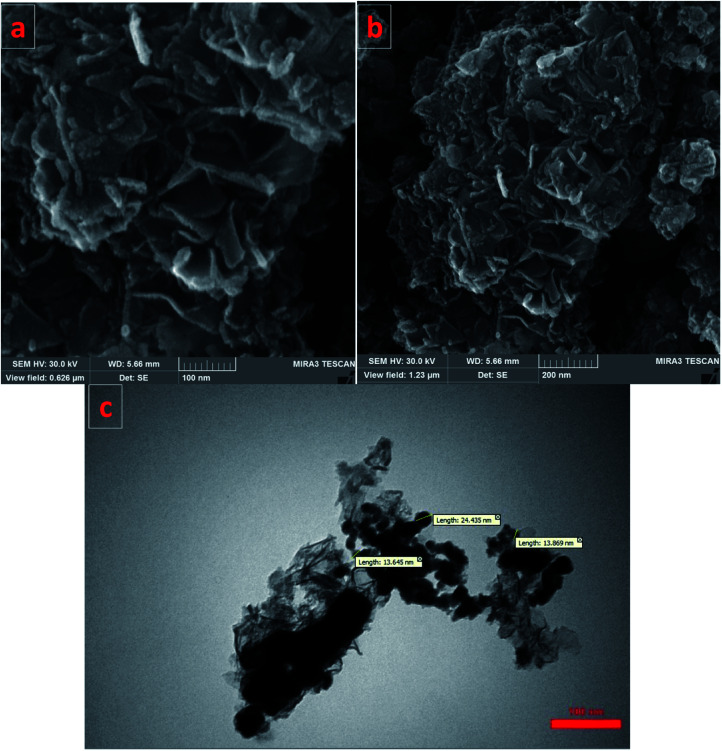
SEM images of Fe/Ni@RH-SiO_2_ (a and b), and TEM image of Fe/Ni@RH-SiO_2_ (c).

SEM images of the Fe/Ni@RH-SiO_2_ catalyst are displayed in Fig. 4a and b. The amorphous hump after the thermal treatment shows that the rice husk is composed mostly of amorphous silica, but still contains quartz as crystalline form of SiO_2._ Transmission electron-microscopy (TEM) studies of the Fe/Ni@RH-SiO_2_ nanocomposite confirm that the Fe/Ni nanoparticles were successfully incorporated into RH-SiO_2_ support (Fig. 4c). In addition, the particles are distributed in range of 13–24 nm for Fe/Ni@RH-SiO_2_ nanocomposite.

**Fig. 5:**
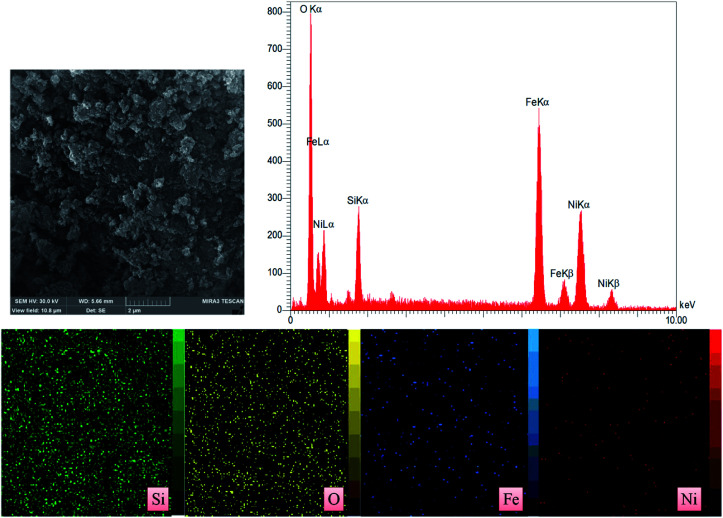
EDX spectrum and X-ray atomic map of Fe/Ni@RH-SiO_2_ nanocomposite.

The EDX pattern (Fig. 5) of the Fe/Ni@RH-SiO_2_ shows the existence of Si, O, Fe and Ni in nanocomposite. Also, the elemental mapping further confirmed a homogeneous distribution of the elements in the RH-SiO_2_. In order to determine the exact amount of Fe and Ni load on the RH-SiO_2_, the ICP-OES analysis was performed and they were found to be 0.44 and 0.49 mmol g^−1^ respectively.

**Fig. 6:**
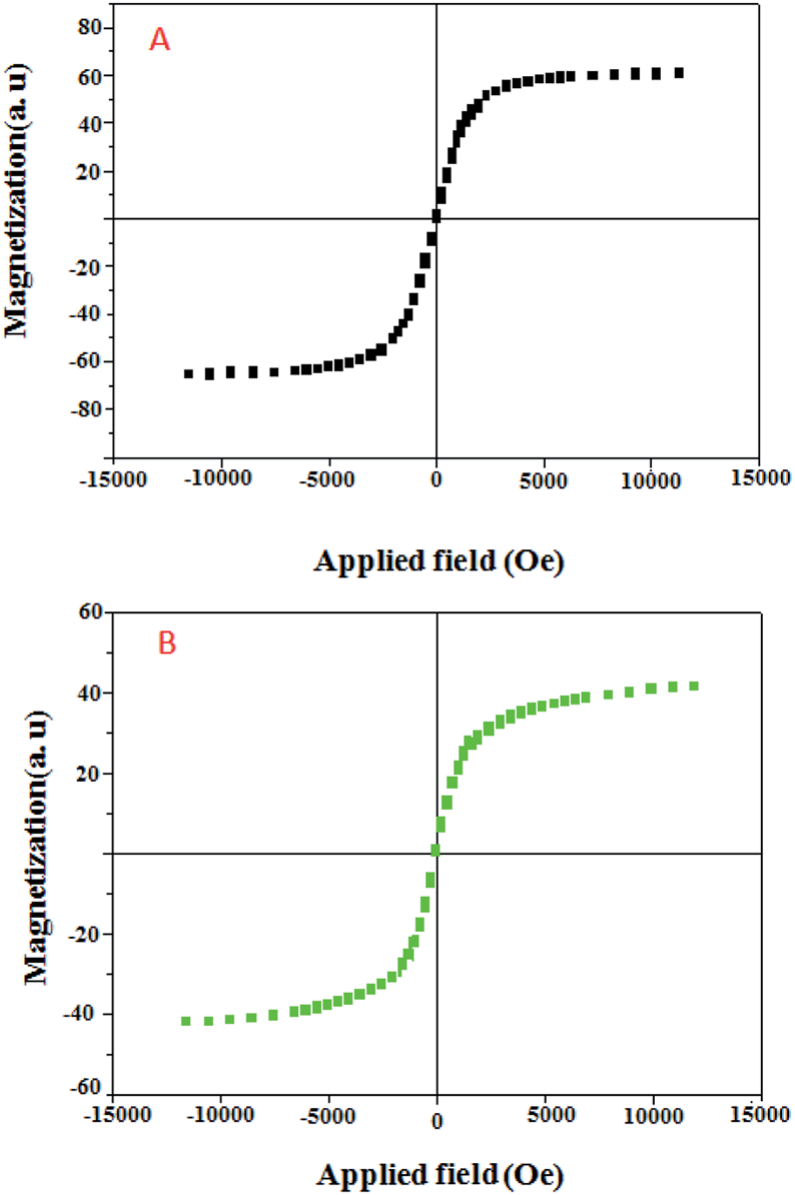
Magnetization curves of Fe@RH-SiO_2_ (A), and Fe/Ni@RH-SiO_2_ (B).

The magnetic properties of the Fe@RH-SiO_2_ (A) and Fe/Ni@RH-SiO_2_ (B) nanomaterials were analyzed using sample magnetometer analysis (Fig. 6). It should be mentioned that the saturation magnetization value (*M*_s_) of the bare Fe@RH-SiO_2_ is 60.10 emu g^−1^ while after coating of Ni nanoparticles, *M*_s_ value of Fe/Ni@RH-SiO_2_ is 40.91 emu g^−1^. However, this magnetic property of Fe/Ni@RH-SiO_2_ is still enough to ensure that the catalyst can easily be recovered from the solution using an external magnet force.

**Fig. 7:**
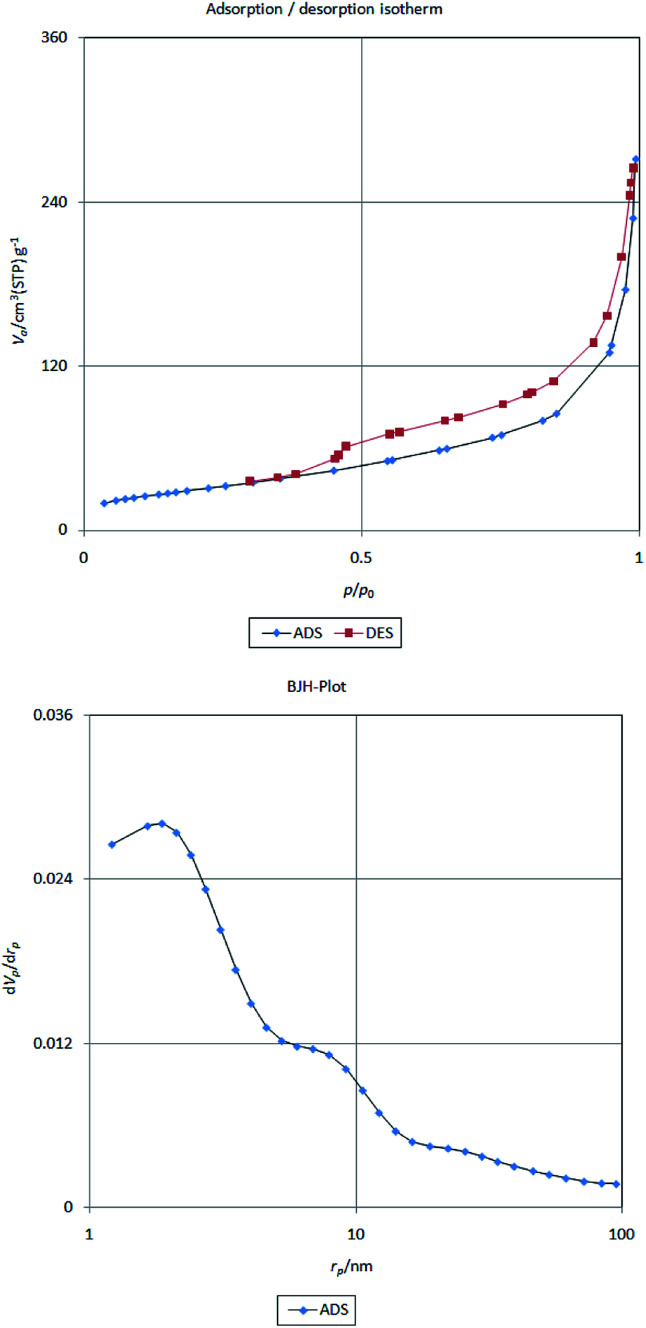
Nitrogen adsorption/desorption isotherm (top) and pore size distributions curve (bottom) of Fe/Ni@RH-SiO_2_

The surface area, the pore volume and the distribution of pore size of Fe/Ni@RH-SiO_2_ was also determined using BET adsorption isotherm and Barrett–Joyner–Halenda (BJH) techniques (Fig. 7). The catalyst had type IV isotherm, indicating that the mesostructure remained intact. According to the results in Table 1 surface area, pore volume and pore size of Fe/Ni@RH-SiO_2_ (129.42 m^2^ g^−1^, 3.84 cm^3^ g^−1^ and 14.33 nm, respectively), decreased compared with Fe@RH-SiO_2_ and RH-SiO_2_,^70^ indicating the Fe/Ni nanoparticles are successfully loaded onto the silica matrix.

**Table 1:** Textural properties of the RH-SiO_2_ and the Fe/Ni@RH-SiO_2_

Samples	BET surface area [m^2^ g^−1^]	Pore volume [cm^3^ g^−1^]
RH-SiO_2_	347	10.4
Fe@RH-SiO_2_	190.7	7.33
Fe/Ni@RH-SiO_2_	129.42	3.84

**Fig. 8:**
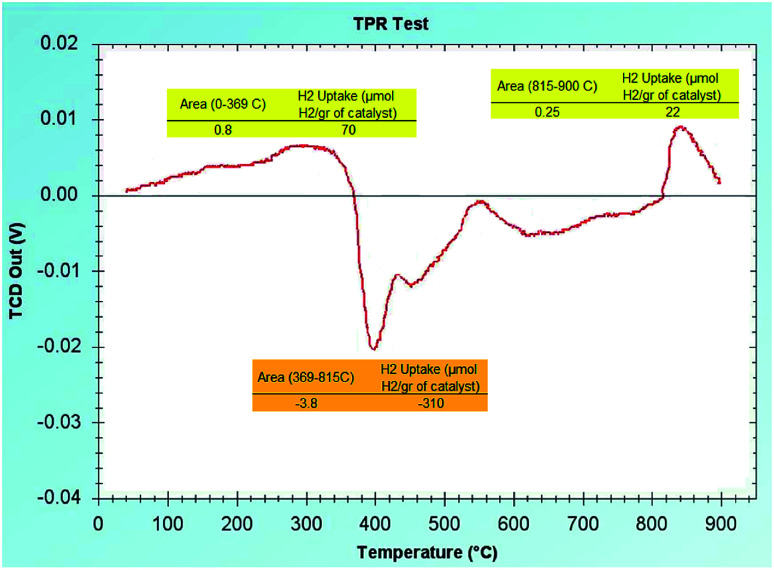
Temperature programmed reduction of Fe/Ni@RH-SiO_2_

To show the oxidation state of the available Ni and Fe in the RH-SiO_2_ support, we have analyzed the (H_2_-TPR) graph. As shown in Fig. 8, there are small picks visible in the positive region of the graph with small percentage which indicate that small percentages of Fe(ii) and Ni(ii) have remained on the RH-SiO_2_ which have not been reduced. Also, a pick is visible on the negative region of the graph in the (400 °C) area which is an indication of H_2_ release/expulsion. Therefore, it can be concluded that the metals have been reduced on the RH-SiO_2_ with NaBH_4_.

### 3.2 Catalytic activity

Initially, we proceeded to optimize the catalytic conditions by studying the reduction of nitrobenzene. The effect of several different reaction conditions such as the solvent, reducing agents, amount of catalyst and temperature were studied (Table 2).

**Table 2:** Optimization experiments for the reduction of nitrobenzene, using Fe/Ni@RH-SiO_2_ nanocomposite

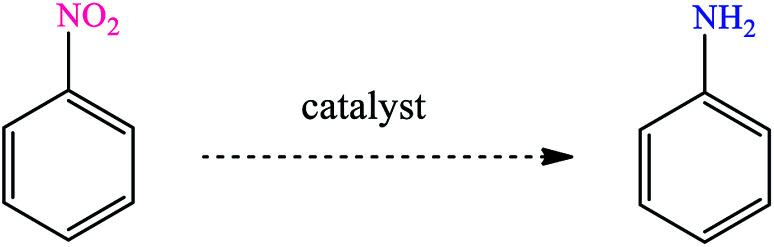						
Entry	Catalyst amount (mg)	Reducing agent	Temp. (°C)	Solvent	Time (min)	Yield^a^ (%)
1	30	NaBH_4_ (4 mmol)	50	CH_3_CN	120	—
2	30	NaBH_4_ (4 mmol)	50	*n*-Hexane	140	—
3	30	NaBH_4_ (4 mmol)	50	Toluene	100	—
4	30	NaBH_4_ (4 mmol)	50	CH_2_Cl_2_	100	—
5	30	NaBH_4_ (4 mmol)	50	EtOAc	100	40
6	30	NaBH_4_ (4 mmol)	50	H_2_O	10	97
7	30	NaBH_4_ (4 mmol)	50	EtOH	45	77
8	—	NaBH_4_ (4 mmol)	50	H_2_O	100	—
9	30	NaBH_4_ (4.5 mmol)	50	H_2_O	10	97
10	30	NaBH_4_ (3 mmol)	50	H_2_O	45	85
11	30	NaBH_4_ (2 mmol)	50	H_2_O	45	79
12	30	NaBH_4_ (1 mmol)	50	H_2_O	45	68
13	30	—	50	H_2_O	100	—
14	20	NaBH_4_ (4 mmol)	50	H_2_O	45	89
15	30	NaBH_4_ (4 mmol)	r.t.	H_2_O	120	35
16	Fe@RH-SiO_2_	NaBH_4_ (4 mmol)	50	H_2_O	90	70
17	30	H_2_ (1 atom)	100	H_2_O	180	37
18	30	HCOOH	60	H_2_O	30	51
19	30	^i^PrOH	100	—	480	60

a Isolated yield.

To investigate the best reaction temperature, this procedure was studied at various temperatures and the best one was 50 °C (Table 2, entry 6). This procedure was studied in different solvents such as EtOH, H_2_O, CH_2_Cl_2_, *n*-hexane, toluene and EtOAc and it was found that H_2_O is a suitable solvent for this reaction and with the nonpolar solvent under the same condition no reaction to proceeded (Table 2, entries 1–4). Then, the different reducing agents HCOOH, ^i^PrOH and H_2_ were studied (Table 2, entries 17–19). These hydrogen sources showed lower activity compared to that of NaBH_4_. Table also shows that the reaction did not perform in the absence of NaBH_4_ and gave a low yield in a low amount of NaBH_4_ (Table 2, entries 10–13). The reduction NO_2_ group did not performed in the absence of catalyst and the reaction yields were decreased in the low amounts of catalyst (Table 2, entries 8 and 14). Furthermore, the reaction was performed in the presence Fe@RH-SiO_2_ as catalyst, but it did not proceed after a long time (Table 2, entry 16). This result confirms that optimum reaction conditions to afford aniline as a sole product in high yield was the molar ratio of 1 : 4 for nitrobenzene and NaBH_4_ in the presence of 0.03 g of RH@Fe/Ni catalyst.

After finding to improve the reaction conditions, we employed different substituted nitroarenes for the synthesis of arylamines. The results indicated that both electron releasing, and electron withdrawing groups have good to excellent yields in short time without the formation of any byproducts (Table 3). Molecules with the complexity of aldehyde and nitro groups did not display any selectivity and both of mentioned functional groups were reduced with the same reactivity (Table 3, entries 6 and 8). Also, Primary aliphatic nitro compounds were rapidly reduced to their corresponding amines in excellent yields (Table 3, entries 10 and 11).

**Table 3:** Reduction of nitro compounds by NaBH_4_ in the presence of Fe/Ni@RH-SiO_2_ catalyst in H_2_O at 50 °C as green condition

Entry	Nitro compound	Product	Time (min)	Yield^a^ (%)
1	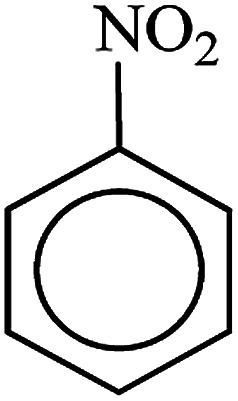	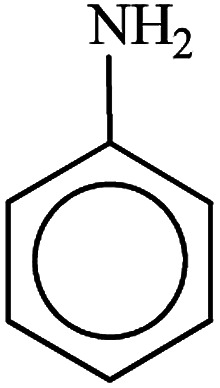	10	97
2	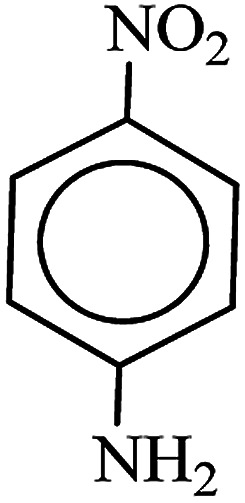	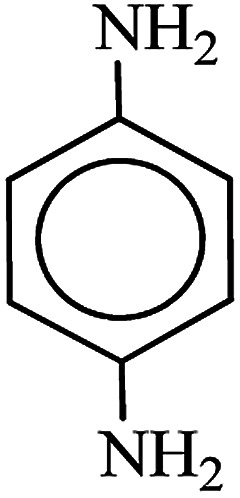	10	95
3	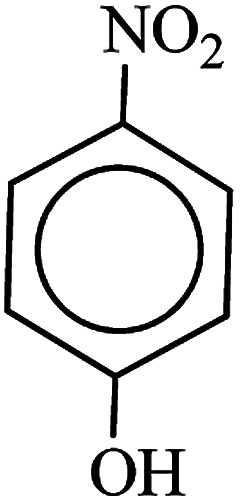	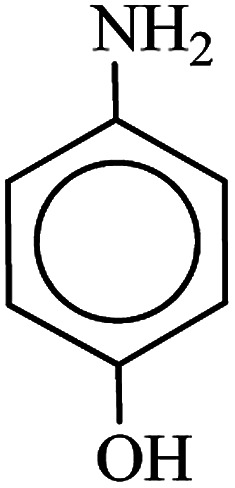	12	93
4	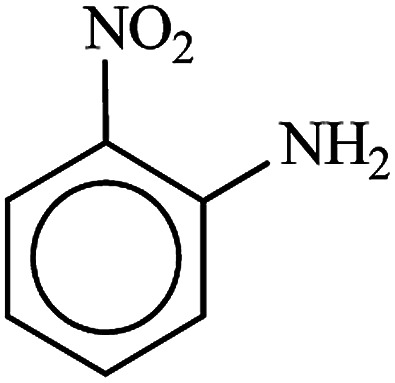	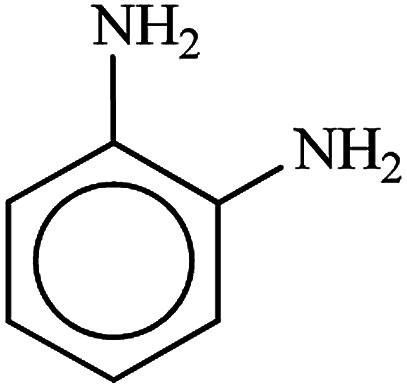	10	90
5	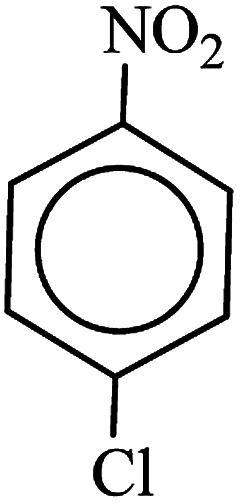	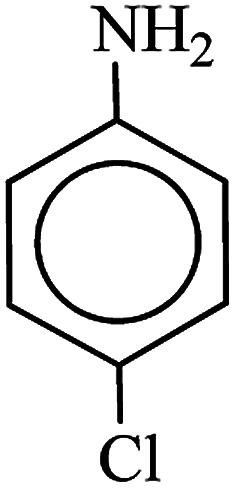	15	91
6	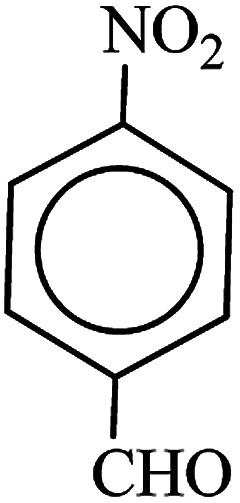	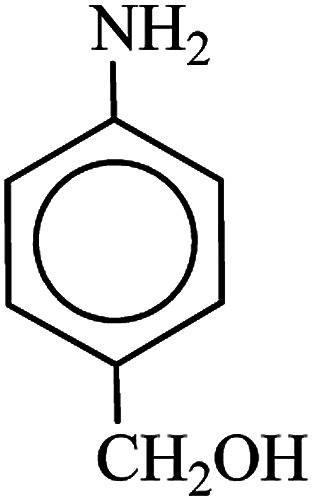	8	95
7	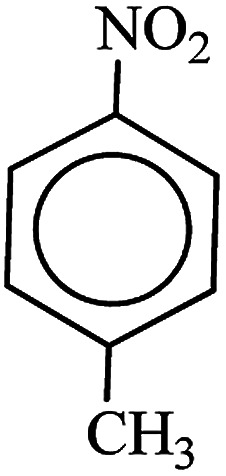	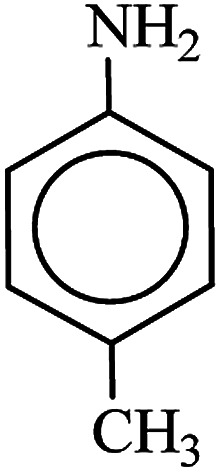	10	94
8	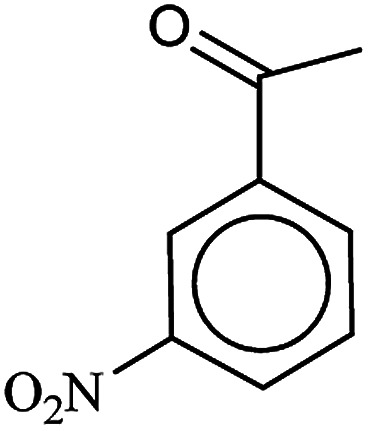	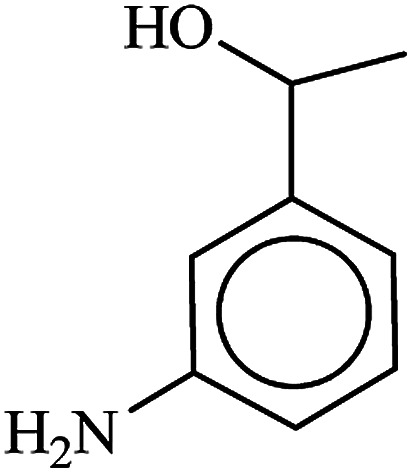	7	92
9	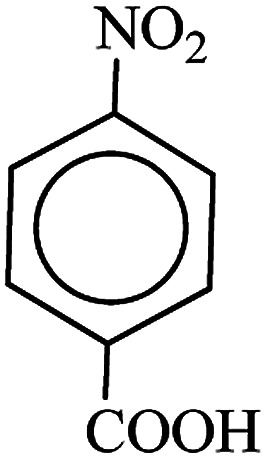	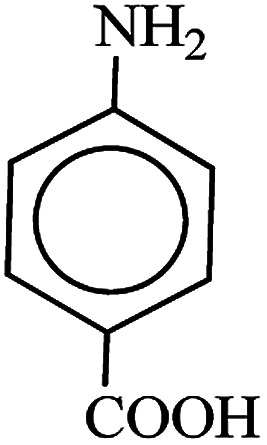	12	90
10	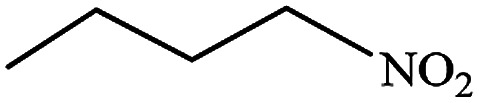	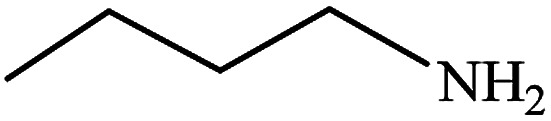	10	93
11	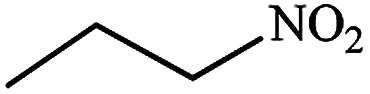	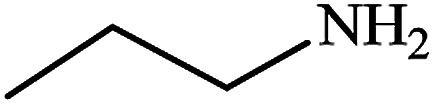	8	95

a Isolated yield.

In comparison with several previously reported methods in the literature, it has been demonstrated that the amorphous silica nanoparticles catalyst provided a high activity and green catalytic protocol for the reduction of nitrobenzene (Table 4). These results clearly show that the catalyst is equally or more efficient to other catalysts reported in terms of easy catalyst isolation and suitable recyclability, high yields, the amount of used catalyst, the reaction temperature and reaction time.

**Table 4:** Comparison of catalytic activity of Fe/Ni@RH-SiO_2_ catalyst with the some recently reported procedures

Entry	Catalyst	Time (min)	Reaction conditions	Yield (%)	Ref.
1	Fe/Ni@RH-SiO_2_	10	NaBH_4_, H_2_O, 50 °C (30 mg)	97	This work
2	Pd-NPs@Oak gum	60	NaBH_4_, EtOH:H_2_O, 50 °C (6.0 mg)	96	71
3	Pt/CoFe-LDH	60	NaBH_4_, isopropanol, 70 °C (10 mg)	97	72
4	Cu/Al_2_O_3_	40–45	NaBH_4_, toluene, 115 °C (200 mg)	99	73
5	Fe_2_O_3_/Ag	30	NaBH_4_, H_2_O, 100 °C, (1 mg)	99	74
6	Zinc phthalocyanine in PEG-400	8	NaBH_4_, EtOH, 100 °C, (1 mmol)	99	75
7	γ-Fe_2_O_3_@HAp-CD·Ag	30	NaBH_4_, H_2_O, 80 °C, catalyst (0.05 g)	98	48
8	Fe–Cu@MCC	8	NaBH_4_/H_2_O, 70 °C, (0.03 g)	93	66

### 3.3 Recycling of Fe/Ni@RH-SiO_2_ catalyst

**Fig. 9:**
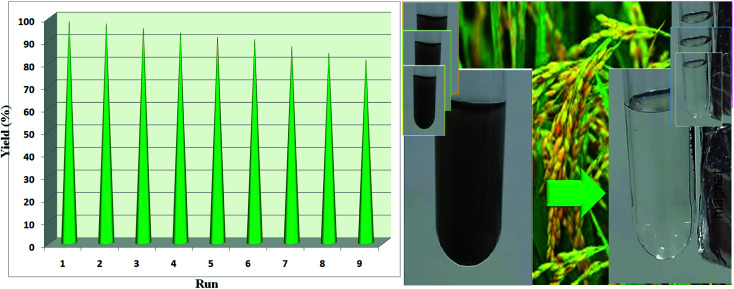
Recyclability of Fe/Ni@RH-SiO_2_ nanocomposite in the reduction of nitrobenzene to aniline.

For studying the recyclability of Fe/Ni@RH-SiO_2_, the catalyst can be recovered from the reaction mixture by an external magnet and washed with Et_2_O and then dried under air atmosphere. The catalyst was recycled and used more than 9 successive runs without considerable decrease in activity (Fig. 9).

**Fig. 10:**
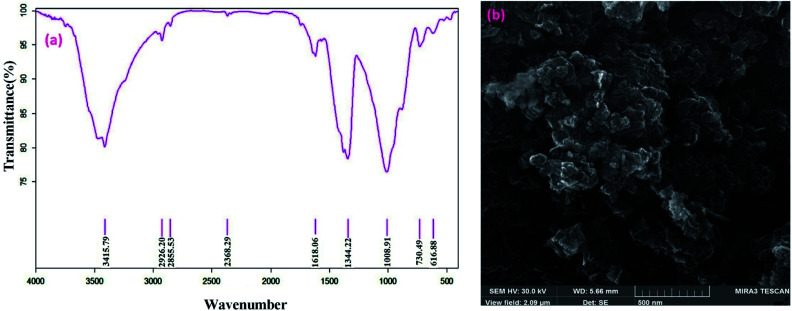
FT-IR spectrum (a) and SEM image (b) of recovered Fe/Ni@RH-SiO_2_ catalyst.

The FT-IR and SEM analyses of the recovered catalyst were carried out following the ninth cycle, and the results are displayed below (Fig. 10). These analyses demonstrated that the chemical structure of the catalyst was remained unchanged.

Final investigation of the recycled catalyst was carried out by measuring the content of the loaded Fe and Ni atoms by ICP-OES technique. It was found that the amounts of Fe and Ni in fresh catalyst and the recycled one after 9 times are 0.46–0.38 mmol g^−1^, and 0.42–0.36 mmol g^−1^ respectively, which there by indicating the minimal amounts Fe and Ni leaching in the catalytic process and affirming the stability and efficiency of the catalyst. Also, the reduction of 4-methylnitrobenzene in the presence of Fe/Ni@RH-SiO_2_ has been investigated by carrying out a hot filtration test in order to find whether the catalyst is truly heterogeneous in nature or whether Ni is leaching out from the solid catalyst to the solution. In this experiment, after continuing the reaction under optimized conditions, we found the yield of product in a half time of the reaction to be 68%. Then the reaction was repeated and in a half time of the reaction, the catalyst was removed from the reaction mixture, using a magnet. The yield of reaction in this stage was 71% that confirmed the reaction proceeds heterogeneously with stability of the catalyst and no significant leaking of effective Ni metal.

## 4. Conclusions

In the present work, magnetic nanoparticles of bimetallic Fe/Ni were immobilized on amorphous RH-SiO_2_ extracted from rice husk. The TEM, X-ray atomic map and XRD analyses successfully confirmed well immobilization and high stability of Fe and Ni moieties onto RH-SiO_2_ framework. The magnetic property of the nanocomposite was investigated by VSM analysis. The catalytic status of nanocomposite was obtained as a green system for the reduction of nitro compounds to corresponding amines in water by using NaBH_4_ as the source of hydrogen.

The key features of this method are high yields, simple operations, time, environmental compatibility, availability and reusability of the catalyst. The reaction conditions (H_2_O as green solvent) coupled with activity and stability of the nanocomposite make the described heterogeneous catalyst highly desirable from the green chemistry point of view. We expect that this feature will enable this protocol to find extensive applications in the field of academic area and pharmaceutical industry.

## Conflicts of interest

There are no conflicts to declare.

## Supplementary Material
